# Multifunctional Mitochondria-Targeting Nanosystems for Enhanced Anticancer Efficacy

**DOI:** 10.3389/fbioe.2021.786621

**Published:** 2021-11-24

**Authors:** Tingting Hu, Zhou Qin, Chao Shen, Han-Lin Gong, Zhi-Yao He

**Affiliations:** ^1^ Department of Pharmacy, State Key Laboratory of Biotherapy and Cancer Center, National Clinical Research Center for Geriatrics, West China Hospital, Sichuan University, Chengdu, China; ^2^ Department of Integrated Traditional Chinese and Western Medicine, West China Hospital, Sichuan University, Chengdu, China; ^3^ Key Laboratory of Drug-Targeting and Drug Delivery System of the Education Ministry, Sichuan Engineering Laboratory for Plant-Sourced Drug and Sichuan Research Center for Drug Precision Industrial Technology, West China School of Pharmacy, Sichuan University, Chengdu, China

**Keywords:** mitochondria, targeted drug delivery systems, liposomes, nanoparticles, nanomicelles

## Abstract

Mitochondria, a kind of subcellular organelle, play crucial roles in cancer cells as an energy source and as a generator of reactive substrates, which concern the generation, proliferation, drug resistance, and other functions of cancer. Therefore, precise delivery of anticancer agents to mitochondria can be a novel strategy for enhanced cancer treatment. Mitochondria have a four-layer structure with a high negative potential, which thereby prevents many molecules from reaching the mitochondria. Luckily, the advances in nanosystems have provided enormous hope to overcome this challenge. These nanosystems include liposomes, nanoparticles, and nanomicelles. Here, we summarize the very latest developments in mitochondria-targeting nanomedicines in cancer treatment as well as focus on designing multifunctional mitochondria-targeting nanosystems based on the latest nanotechnology.

## Introduction

**FIGURE 1:**
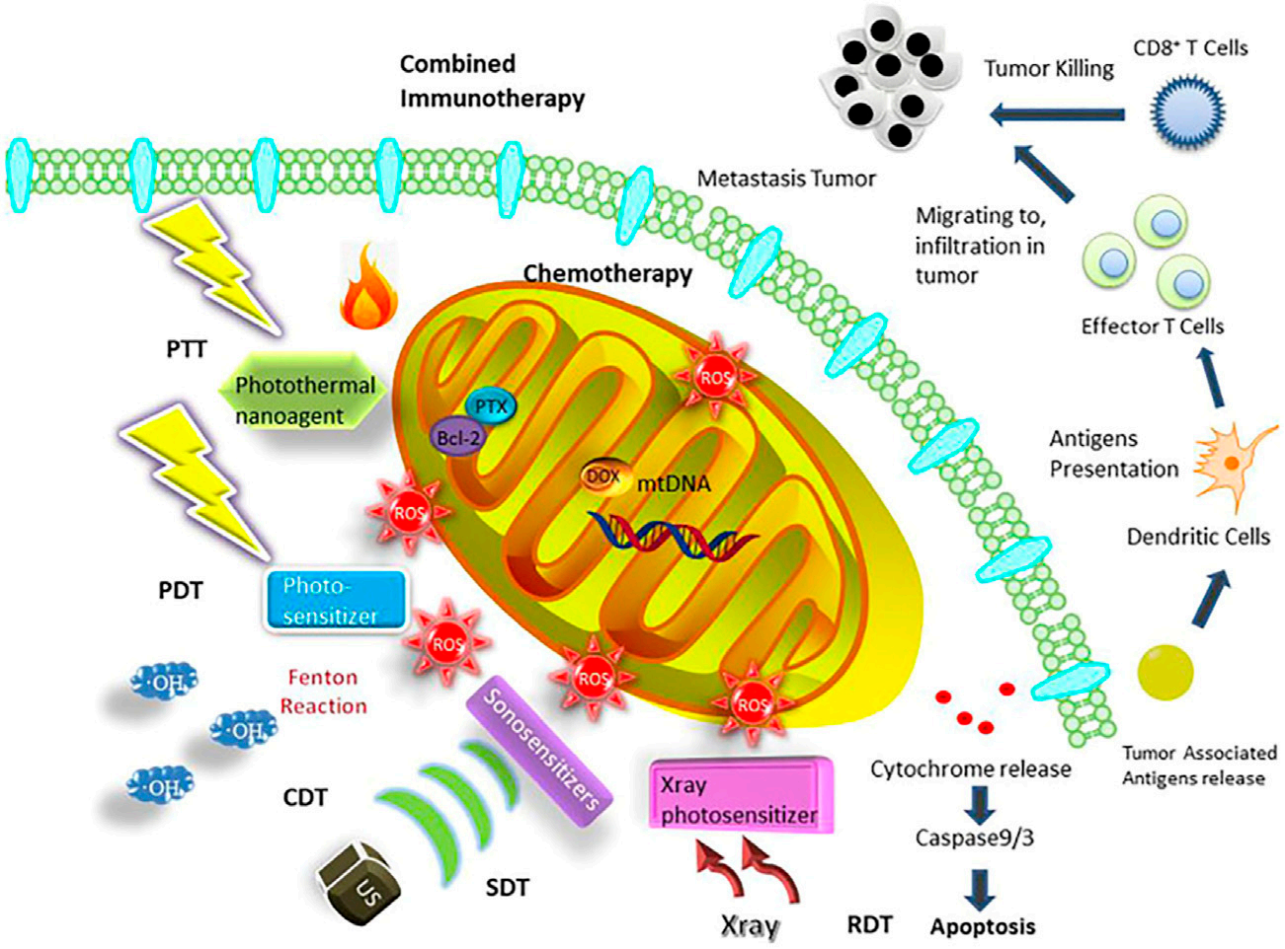
Mitochondria-targeted therapeutic strategies based on nanosystems containing chemotherapy, PDT, PTT, SDT, CDT, RDT, and combined immunotherapy (Gao et al., 2021).

**FIGURE 2:**
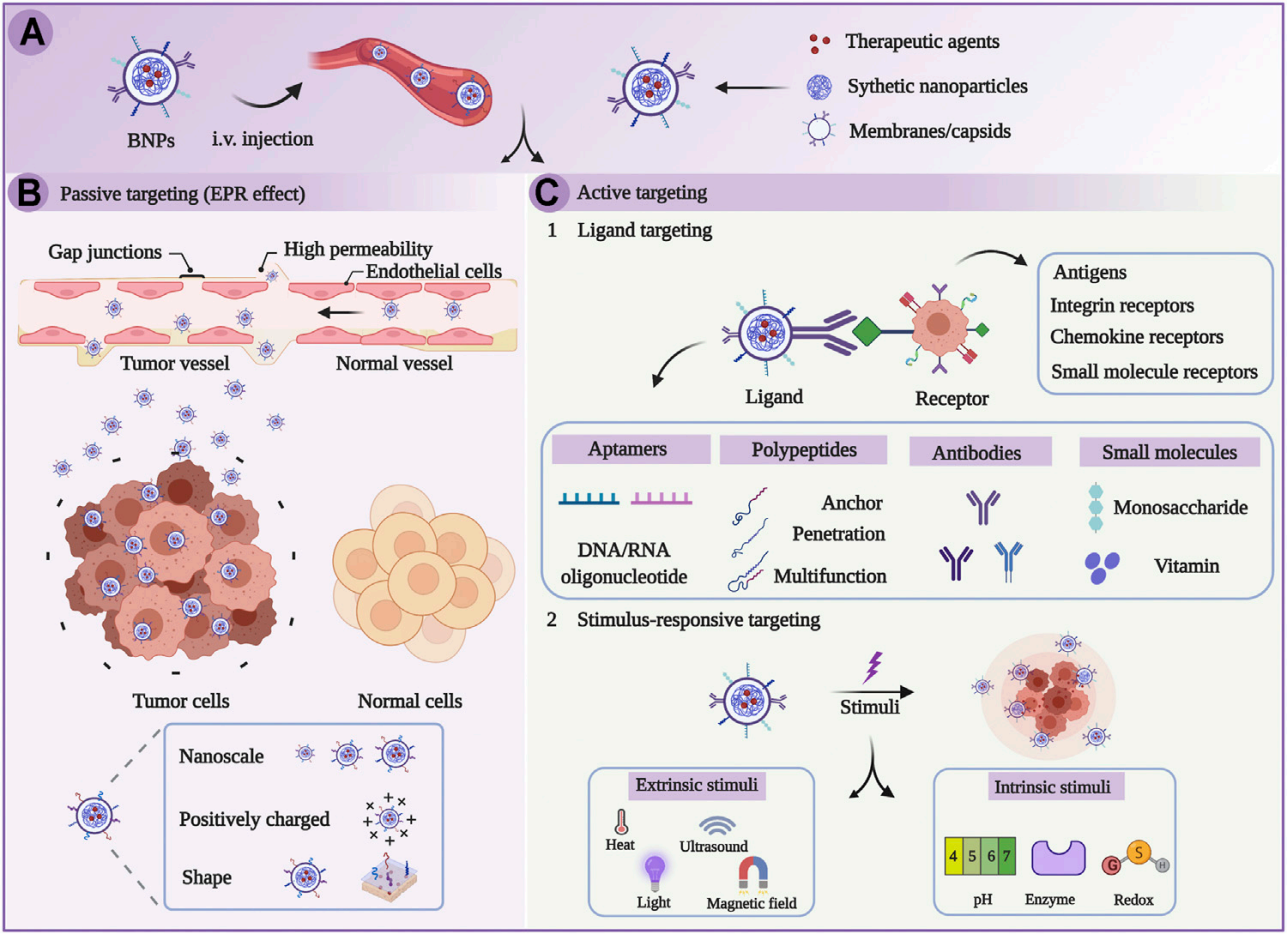
Schematic illustration of targeted therapy on the basis of biomimetic nanoparticles (BNPs). **(A)** BNPs were injected intravenously. **(B)** BNPs passively target tumors through EPR effects. **(C)** BNPs actively target tumors through the stimulus-dependent and ligand-mediated pathway (Chen et al., 2021).

It has been generally recognized that DNA mutations lead to mitochondria disfunction and ultimately lead to various diseases including cancer (Basu et al., 2020; Dhanasekaran et al., 2020; Wang et al., 2020), mainly due to alterations in energy metabolism and the electron transport chain (ETC) system (Pathania et al., 2009; Zong et al., 2016; Chan, 2020; Allemailem et al., 2021). In recent years, numerous studies have shown that mitochondria, a kind of subcellular organelle, play crucial roles in cancer cells as an energy source and as a generator of reactive substrates, which concern the survival, invasion, proliferation, and drug resistance of cancer: (1) Mitochondria are indispensable for cancer survival: Mitochondrial glycolysis pathway on the basis of glutaminolysis promotes the generation of ATP to maintain the survival of tumor cells (Modica-Napolitano and Weissing, 2015; Khaled et al., 2021). In addition, autophagy is an important source of glutamine to regulate the mitochondrial energy metabolism in lung tumors (Strohecker et al., 2013; Guo and White., 2013; White et al., 2015). Furthermore, fatty acids also serve as another substrate for oxidation to facilitate the generation of mitochondrial ATP and consequently support the tumor survival (Carracedo et al., 2013; Lin et al., 2017; Liu et al., 2021). (2) Mitochondria facilitate tumor metastasis and invasion: Tumor metastasis and invasion primarily rely on the oxidative phosphorylation of mitochondria. In this process, the peroxisome-proliferator regulator plays a crucial role in mitochondrial function and biogenesis by triggering the oxidative phosphorylation of mitochondria, which thereby stimulates the metastasis and invasion of the cancer cells (LeBleu et al., 2014; Chen et al., 2016; Liu L. et al, 2021). Energy metabolism allows for the production of reactive oxygen species (ROS), leading to activation of pyk2 and src protein tyrosine kinases, which ultimately stimulates and promotes tumor development and invasion (Xu et al., 2015). In cancer cells, the extra- and intracellular Ca^2+^ pools accumulating in mitochondria influence the permeability of mitochondrial permeability transition pore (mtPTP), which thereby affects tumor cell apoptosis (Qin et al., 2021; Madreiter-Sokolowski et al., 2021). (3) Mitochondria are related to multiple drug resistance: Genotoxic drugs trigger mitochondrial shift by means of regulating mitochondrial energy metabolism and upregulating mitochondrial oxidative phosphorylation, which results in drug resistance to chemotherapy (Lee et al., 2017; Lee et al., 2018). Researchers have confirmed that BRAF inhibitors trigger the oxidative phosphorylation of mitochondria, resulting in ROS generation in tumor cells. Mitochondrial oxidative metabolism may be another mechanism that reduces the anticancer effect of BRAF inhibitors (Haq et al., 2013). (4) Mitochondria are in charge of energy metabolism and tumor cell proliferation: Recent studies have illustrated that tumor cells could promote the generation of glutamine by oxidizing glucose-derived pyruvate through the pyruvate dehydrogenase (PDH)-dependent pathway in mitochondria, which is crucial for tumor growth (Woolbright et al., 2019; Khan et al., 2020; Nie et al., 2020). Therefore, precise delivery of anticancer agents to mitochondria can be a novel strategy for enhanced cancer treatment (Weinberg and Chandel, 2015; Vasan et al., 2020; Guo et al., 2021; Gao et al., 2021; Zhu et al., 2021). Nowadays, various therapeutic strategies, such as chemotherapy, photodynamic therapy (PDT), photothermal therapy (PTT), sonodynamic therapy (SDT), chemodynamic therapy (CDT), and combined immunotherapy based on mitochondria targeting nanoplatforms, have been applied to achieve better therapy efficacy (Figure 1, from Gao et al., 2021). However, mitochondria have a four-layer structure with a high negative potential (−160 to −180 mV), which thereby prevents many molecules from reaching the mitochondria (Frey and Mannella, 2000; Tait and Green, 2010; Battogtokh et al., 2018; Gao et al., 2021). Luckily, the development of nanotechnology has provided the great potential to overcome the membrane barriers. Multifunctional nanosystems can further improve the selectivity as well as enhance the efficacy of anticancer agents (Figure 2), considering the non-functionalized nanosystems’ drawbacks such as poor tumor cell targeting, rapid clearance during circulation, poor endosome/lysosome escape, and nonspecific toxicity (Chi et al., 2017; Khatun et al., 2017; Liu et al., 2018; Song et al., 2018; Wang et al., 2019; Zhang et al., 2020; Chen et al., 2021). Here, we summarize the very latest developments in mitochondria-targeting nanomedicines in cancer treatment as well as focus on designing multifunctional mitochondria-targeting nanosystems based on the latest nanotechnology.

## Mitochondria Targeting With the Aid of Multifunctional Nanosystems

### DQAsomes

Dequalinium (DQA), a kind of mitochondria-specific targeting ligand with two delocalized cation centers, can self-assemble into liposome-like cationic vesicles known as DQAsomes (Horobin et al., 2007). DQAsomes are the earliest reported mitochondria-targeting nanoplatforms for delivery of plasmid DNA (pDNA) (D'Souza et al., 2003). DQAsomes could selectively deliver pDNA to mitochondria by taking advantage of its positive charge property. In recent years, DQAsomes have also been applied to load chemotherapy drugs such as docetaxel and curcumin. Nevertheless, low transfection efficiency, short half-life, and potential toxicity limit their further application.

DQAsomes have been applied to precisely deliver many agents into the mitochondria without any off-target leakage (D'Souza et al., 2005; Lasch et al., 1999). However, considering that short half-life is the major disadvantage for naked DQAsomes and positively charged DQAsomes may trigger nonspecific toxicity, shielding the positively charged DQAsomes and lengthening the half-life in blood are great challenges for constructing a new-type DQAsome delivery system (Chi et al., 2017; Khatun et al., 2017; Liu et al., 2018; Song et al., 2018; Wang et al., 2019; Zhang T. et al., 2020). Shi et al. (2018) synthesized a HER-2 peptide-modified and tumor acidic microenvironment-sensitive PEG derivate [HER-2 peptide-PEG_2000_-Schiff base-cholesterol (HPSC)] and applied it to shield naked DQAsomes to get HPS-DQAsomes. In their study, doxorubicin (DOX)-loaded HPS-DQAsomes with active cancer cell targeting ability, pH-responsive PEG leakage, and mitochondrial targeting ability endowed by HER-2 peptide-PEG_2000_-Schiff base-cholesterol (HPSC) and DQA induced the apoptosis of MCF-7/ADR cancer cells by reducing the membrane potential of mitochondria, triggering the release of cytochrome c, and inducing the cascade of caspase-3 and -9, which in turn confirmed that HPS-DQAsomes could be a promising nanocarrier to overcome MDR.

### Liposomes

Liposomes are tiny bilayer vesicles formed by phospholipids dispersed in water, which are generally biocompatible. Inspired by the success of DQAsomes, many liposomes systems based on the mitochondria-targeting ligands, such as TPP and STPP, have been constructed for mitochondrial targeted delivery. However, cellular toxicity of targeted liposomes is still unknown (Durazo and Kompella, 2012).

_D_-(KLAKLAK)_2_ (KLA), a positively charged, mitochondria-specific targeting peptide, has been confirmed to be a good targeting ligand for mitochondria and can damage the mitochondrial membrane as long as a threshold concentration (10 μmol) is reached. RGD (Arg-Gly-Asp) can effectively target vascular endothelial cells. Therefore, Sun et al. (2017) synthesized KLA-PEG_2000_ modified DSPE (KLA-PEG_2000_-DSPE) and cRGD-PEG_2000_ modified DSPE (cRGD-PEG_2000_-DSPE) as mitochondria- and vascular endothelial cell-targeting moieties, respectively. Then, sequentially tumor-targeting liposomes including these two targeting peptide-modified lipids were constructed to loading paclitaxel (PTX) to get PTX-loaded therapeutic liposomes (RGD-KLA/PTX-Lips). In their study, KLA and cRGD showed a cooperative effect in promoting the cellular uptake, which significantly lowered the half-maximal inhibitory concentration (IC50) value of RGD-KLA/PTX-Lips with 4T1 cells and human umbilical vascular endothelial cells (HUVECs). The strong tumor growth inhibition (80.6%) and remarkable antineovascularization effects with no systemic toxicity were also achieved in RGD-KLA/PTX-Lips-treated 4T1 tumor-bearing BALB/c mice.

K peptide, another mitochondria-targeting peptide with a positive charge, can increase nanoparticle uptake into mitochondria and decrease mitochondrial membrane potential, trigger ATP depletion, contribute to cancer cell apoptosis, and consequently avoid MDR. RF peptide, a potent cell-penetrating peptide (CPP), can strengthen the intracellular uptake and facilitate the endosomal escape of nanoparticles. H peptide, isolated by phage display for binding to nerve/glial antigen 2 (NG2), has been proven to effectively target tumor neovasculature. Juang et al. (2019) applied those three peptides to decorate pH-sensitive solid lipid nanoparticles (SLN) and pH-sensitive liposomes, and then miR-200 and irinotecan were loaded into those multifunctional SLN and liposomes, respectively, to obtain miR-200/omSLN-RFKH and Iri/omLip-RFKH. In their study, these specially designed nanoparticles exhibit pH-responsive release, synergistic effect in enhancing internalization, intracellular distribution, and mitochondrial localization in acidic pH of HCT116 cells. Under the combined treatment of Iri/Lip-RFKH and miR-200/SLN-RFKH, CRC cell apoptosis was observably enhanced and the anticancer effect was observably improved in colon tumor-bearing mice by modulating the EMT/apoptosis/MDR/β-catenin signaling pathways and suppressing the expression of cyclin D1, β-catenin, Rac-1, ZEB1, KRAS, P-gp, p-GSK-3β, Slug, c-Myc, MRPs, and Vimentin.

Feng et al. (2021) constructed a smart, dual-functional liposome delivery system having characteristics of both pH-responsive charge reversal and mitochondria targeting to facilitate agent accumulation in mitochondria and inhibit the growth of tumor cells. Briefly, L-lysine acted as a linker to link 2,3-dimethylmaleic anhydride (DMA) and 1,2-distearoyl-sn-glycero-3-phosphoethanolamine (DSPE) to synthesize a pH-responsive lipid derivative (named DLD). Then, the DLD was mixed with other lipids (Soy lecithin, cholesterol, DSPE-mPEG_2000_) to fabricate charge-reversed, mitochondria-targeting liposomes (named DLD-Lips). At pH 7.4, DLD-Lips were negatively charged; however, the surface charge of DLD-Lips was transformed from negative to positive, when DLD-Lips accumulated in a weakly acidic tumor microenvironment (TME). After treatment with hyperoside (HYP)-loaded DLD-Lips (HYP/DLD-Lips), the growth of CBRH-7919 tumor cells was significantly inhibited, and the antitumor effect (tumor growth inhibition, 88.79%) was observably enhanced in CBRH-7919 cell-bearing BALB/C mice.

### Inorganic Nanoparticles

Inorganic nanoparticles made from inorganic components such as graphene oxide, platinum, gold, silica, and carbon are ideal vehicles for targeted drug delivery, due to their ideal size, good stability, biocompatibility, high drug loading capacity, and easy modification of targeting moieties for mitochondria targeting. However, there are still many challenges in clinical transformation of inorganic nanoparticles. The biggest challenge is long-term toxicity *in vivo*, and whether they can be removed from the body remains to be proven (Hofmann et al., 2015; De Matteis et al., 2017; Yang et al., 2019). It is known that hypericin (HY), a natural compound extracted from *Hypericum perforatum* L., is both an antitumor photosensitizer and a tumor-targeting ligand (Liu et al., 2015). Han et al. (2018) synthesized HY-modified graphene oxide derivatives (named GO-PEG-SS-HY) and then used them to load DOX (named GO-PEG-SS-HY/DOX), which not only had the advantage of synergistic photo- and chemo-therapeutic effects endowed by HY and DOX, respectively, but also could effectively deliver DOX to mitochondria with the aid of HY. After combined treatment with laser irradiation and GO-PEG-SS-HY/DOX, the apoptosis in both MDA-MB-231 and MCF-7 cells was significantly enhanced and the antitumor efficacy was remarkably amplified in MDA-MB-231 cell-bearing mice by modulating the mitochondrial-mediated apoptosis (MMA) pathway (facilitating the release of cytochrome c and triggering the cascade of caspase-9, caspase-7, caspase-3, and PARP).

**FIGURE 3:**
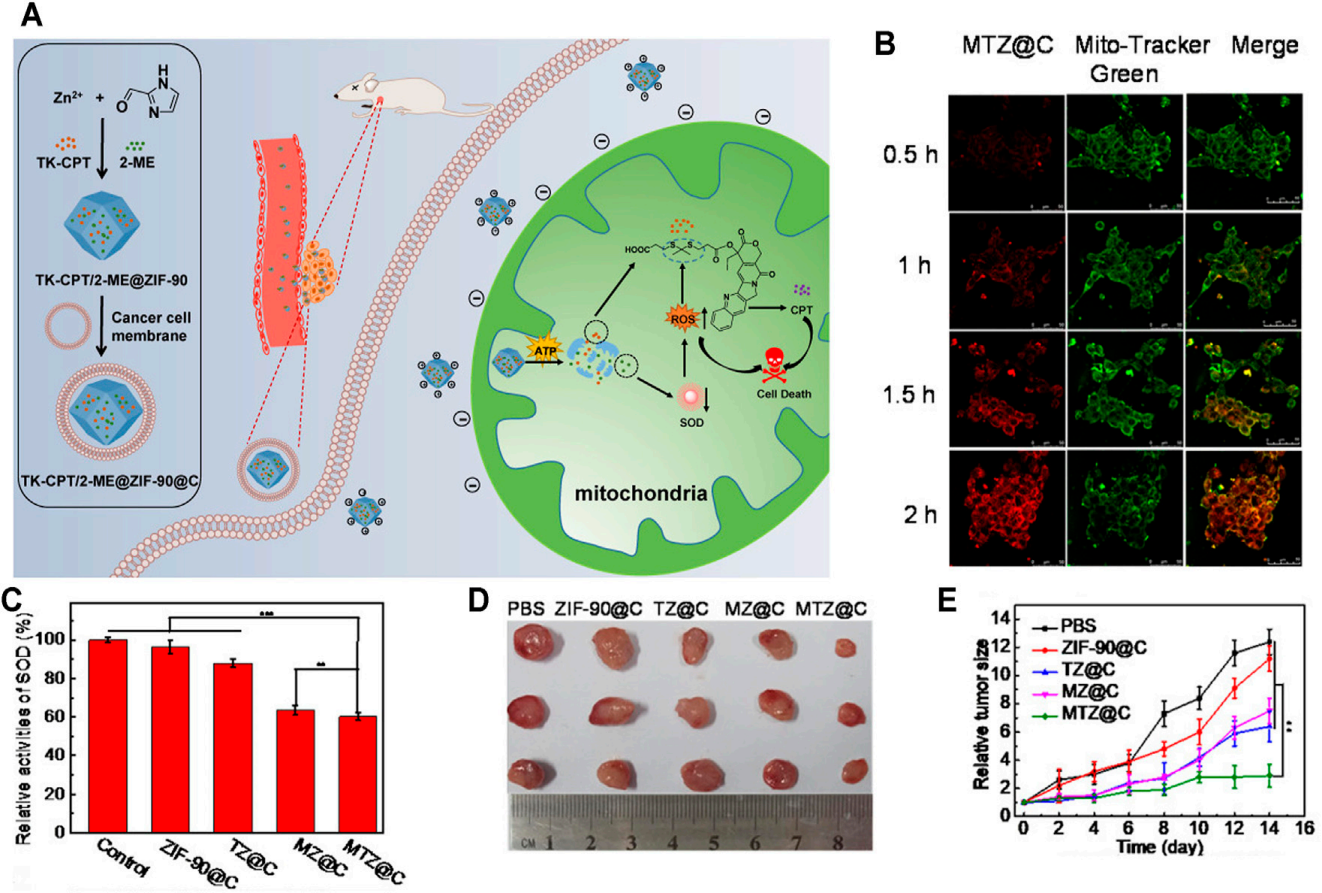
**(A)** Schematic illustration of the preparation of ATP-triggered 2-ME/TK-CPT@ZIF-90@C (MTZ@C) nanoparticles to initiate mitochondrial cascade reactions. When MTZ entered mitochondria, 2-ME was released owing to the ATP-triggered ZIF-90 decomposition. Then, 2-ME amplified the level of ROS in mitochondria, thus facilitating the effective release of parent CPT. **(B)** MTZ@C mitochondrial targeting ability. **(C)** SOD activity inhibition of 2-ME. **(D)** Dissected tumor images after administration for 2 weeks. **(E)** Tumor growth curves within 2 weeks after administration (Pan et al., 2021).

Chemotherapy is the most traditional treatment to cancer therapy; however, low bioavailability and serious adverse reactions limit the clinical applications of some chemotherapy drugs. Therefore, it is urgently needed to develop new effective strategies to deliver the drugs into the mitochondria in a controlled manner. Pan et al. (2021) designed an ATP-triggered, zeolitic imidazole framework-90 (ZIF-90)-based nanosystem to trigger mitochondrial cascade reactions for precise and enhanced cancer therapy (Figure 3A). Firstly, they chose ZIF-90, a compound combined imidazolate-2-carboxyaldehyde and Zn^2+^ as the drug carrier, which has been widely used in drug delivery (Alsaiari et al., 2018; Yang et al, 2019c; Xiao et al., 2020). Due to the positive charges, ZIF-90 can actively target mitochondria. More importantly, ZIF-90 can be disassembled by mitochondrial ATP to liberate the loaded drugs because of the competitive coordination between ATP and Zn^2+^ (Lu et al., 2021). Secondly, they chose both 2-Methoxyestradiol (2-ME) and thioketal-linked camptothecin (TK-CPT, a prodrug) as the drugs. Among them, 2-ME could able to elevate the level of ROS in cancer cells by inhibiting superoxide dismutase (SOD) (Golab et al., 2003), which is essential to increase the oxidative stress and thereby facilitate the effective liberation of free CPT from TK-CPT (Xu et al., 2016). Lastly, the TK-CPT and 2-ME loaded ZIF-90 nanoparticles (2-ME/TK-CPT@ZIF-90, MTZ@C) were coated with a cancer cell membrane to achieve tumor tissue targeting. When MTZ@C specifically reached mitochondria with the aid of ZIF-90, 2-ME was released owing to the ATP-triggered ZIF-90 decomposition. Then, 2-ME amplified the level of ROS in mitochondria, thus facilitating the effective release of parent CPT. Eventually, this cascade of reactions in mitochondria contributed to sustaining high oxidative stress and achieved excellent therapeutic effects both *in vitro* and *in vivo*. As shown in Figure 3B, the red fluorescence of 4T1 cells enhanced gradually with the extension of culture time, indicating that MTZ@C nanoparticles accumulate in mitochondria gradually. In addition, there was a good overlap between the green fluorescence and red fluorescence, indicating that MTZ@C nanoparticles could specifically target the mitochondria. Compared with the groups without 2-ME (PBS, ZIF-90@C, TZ@C), the 2-ME inhibitor groups (MTZ@C, MZ@C) had a significant inhibition on SOD, and the relative activity of SOD decreased by 49.8% and 47.5%, respectively (Figure 3C). Inspired by the *in vitro* therapeutic effect of MTZ@C, Pan et al. evaluated the *in vivo* anticancer effect of MTZ@C in 4T1 cell-bearing mice. As shown in Figure 3D, tumors in the MTZ@C treatment group were significantly smaller than those in other groups, and the tumor growth curve of MTZ@C treatment group (Figure 3E) once again proved that MTZ@C could effectively inhibit the growth of solid tumors.

Although gold nanoparticles have been confirmed to be excellent drug delivery platforms for many drugs, they are not desirable vehicles of PTT agents because of their poor photothermal conversion efficiency and their poor endosome/lysosome escape capability (Feng et al., 2017; Zhang et al., 2019). Herein, Ning et al. (2020) designed a photothermal therapeutic “shell-core” nanosystem CCM-TPP-GNA, where a mitochondrial targeting gold nanoparticle assembly (TPP-GNA) served as the core and PC-3 cancer cell membrane (CCM) served as the shell for enhanced endosome/lysosome escape. In their study, the gold nanoparticles were endowed with mitochondria-targeting ability, excellent absorption, and efficient photothermal conversion capacity in the NIR region by the decoration with synthesized TPP derivatives. The CCM shell facilitated the TPP-GNA to overcome the tumor cell membrane barrier *via* a homotypic membrane fusion process, rather than being stuck in endosomes/lysosomes (Nie et al., 2020). Combined with laser treatment, this synergic “small targeting molecule and biomembrane” two-step cooperative nanosystem showed the strongest anti-tumor effect, where all the tumor tissue disappeared, leaving only black scars at the implanted sites on PC-3 cell-bearing mice.

The ROS elimination by the high concentration of GSH in cancer cells (Bansal and Simon, 2018; Wang J. Y. et al., 2020) and the severe skin photosensitivity during the PDT process (Zhang et al., 2016; Jiang et al., 2019) seriously limit the antitumor efficacy and clinical application of PDT. Huang et al. (2020) constructed a mitochondrial-targeting and pH-triggered hybrid supramolecular photosensitizer by the host–guest interaction, where sodium carboxylatopillar[5]arene (SP[5]A) and (5-carboxypentyl) triphenylphosphonium bromide-modified AuNPs served as the mitochondrial-targeting host motif (named AuTP) and tetra (4-N-valeronitrile pyridyl)porphyrin (TPyP) and cyano-terminated poly(ethylene glycol) (PEG) chains served as the guest motifs, respectively. In their study, this “smart” hybrid supramolecular photosensitizer (named AuTSP) could be turned off by the Förster resonance energy transfer (FRET) effect of AuTP during blood circulation, and AuTSP could be turned on once AuTSP decomposed, owing to the water-soluble, weakly acid pH-sensitive pillar[5]arene on gold nanoparticles and the dynamic property of the host–guest interaction in an acidic TME. More importantly, GSH elimination by AuTP prolonged the lifetime of ROS produced by the PDT near mitochondria and enhanced the PDT antitumor effect further. Overall, this “smart,” mitochondria-targeting, pH-triggered, and self-amplified supramolecular photosensitizer (AuTSP) showed enhanced PDT efficacy, good biocompatibility, and minor side effects both *in vitro* and *in vivo*.

### Polymeric Nanoparticles

Polymeric nanoparticles are self-assembled from amphiphilic blocks or grafted polymers, which are biodegradable, biocompatible, and easily modifiable and possess both hydrophobic cores and hydrophilic shells (Kamaly et al., 2012; Abd Ellah and Abouelmagd., 2017). Due to these outstanding features, polymeric nanoparticles can easily load both hydrophilic and hydrophobic agents and then deliver them to targeted sites. However, their clinical use is strictly controlled, because of the potential toxicity and low immunogenicity (Zielińska et al., 2020).

Triphenylphosphine (TPP), with its high lipophilicity and delocalized positive charge, thus possessing both mitochondrial targeting and mitochondrial damaging functions, is the first small molecule applied to mitochondrial targeting. Till now, TPP has been extensively used to design various mitochondria-targeting nanosystems (Wang R. et al., 2020; Lee et al., 2021; Sun et al., 2021). On the other hand, all-trans retinoic acid (RA), a metabolite of vitamin A, can bind to cellular retinoic acid-binding protein II (CRABP-II) very easily, and this tight-binder exhibits super affinity to the nuclear RA receptor, which allows for the precise nuclear targeting (Majumdar et al., 2011; Zhou, et al., 2017; Ghyselinck and Duester., 2019). Herein, You et al. (2018) constructed “smart, multistage targeted-delivery” polymeric nanoparticles that could co-deliver two different site-oriented prodrugs, prompting them to arrive their targeted nucleus or mitochondria efficiently on the basis of NIR-mediated controlled release. First of all, RA conjugated cisplatin derivative (RA-Pt) was synthesized, where Pt could be delivered to the nucleus of cancer cells with the assistance of RA, facilitating Pt binding to DNA more easily. In the same way, TPP conjugated celastrol derivative (TPP-Cet) was synthesized, where Cet could be efficiently delivered to the mitochondria of cancer cells with the assistance of TPP. Then, these site-oriented prodrugs were loaded into folate and cRGD modified polymeric nanoparticles, allowing for the precise cell membrane targeting, which could maximize the anticancer efficacy and decrease systematic toxicity further. In their study, folate and cRGD dual-targeted and TPP-Cet and RA-Pt loaded nanoparticles (RA-Pt/TPP-Cet@Fc-INPs) showed low cytotoxicity, exhibited significant cancer cell inhibition *in vitro*, and achieved enhanced antitumor efficacy in MCF-7 tumor-bearing mice (tumor growth inhibition, 81.5%).

Polydopamine (PDA), a derivate of dopamine, has been used as a delivery platform owing to its excellent advantages such as easy synthesis, excellent biocompatibility, facile modification, and high drug loading ability (Mrówczyński et al., 2018; Wang et al., 2018; Cheng et al., 2019). Moreover, earlier studies have also confirmed that dopamine could directly modify complex III and complex I of the ETC, thus leading to the destruction of mitochondrial depolarization and mitochondrial respiration (Gluck and Zeevalk, 2004; Bagh et al., 2008; Graves et al., 2020). To promote the penetration of PDA through the mitochondrial inter membrane and enhance the mitochondria-targeting efficiency, Li et al. (2017) synthesized TPP-modified PEG-PDA nanoparticles (PDA-PEG-TPP), where TPP acted as a mitochondrial targeting ligand. In their study, DOX-loaded PDA-PEG-TPP (named PDA-PEG-TPP-DOX) showed excellent ability of reducing drug resistance by downregulating the expression level of pro-caspase 3, which was observed in MDA-MD-231 cancer cells repeatedly treated with PDA-PEG-TPP-DOX.

Considering that cationic nanocarriers have strong cytotoxicity, poor serum stability, and rapid elimination by RES (Chi et al., 2017; Wang et al., 2019; Zhang X. et al., 2020), Fang et al. (2020) synthesized a versatile, mitochondria-targeting, TME-sensitive, and charge-reversal polysaccharide-based nanoplatform. In brief, they constructed a positively charged polymer of gallic acid-chitosan oligosaccharide-dithiopropionate acid-berberine (named GA-CDB), which could actively target the mitochondria. More importantly, GA-CDB was GSH-sensitive, because of the disulfide bond between chitosan oligosaccharide and berberine. GA-CDB eventually self-assembled into Cur-loaded cationic micelles (named GA-CDB@Cur). Then, negatively charged angelica sinensis polysaccharide-phenylboronic acid (AS-PBA) was applied to coat positively charged GA-CDB@Cur. Among them, phenylboronic acid (PBA) can also specifically target the sialic acid epitopes on the tumor surface, which can enhance the tumor cell-targeting capacity. At last, AS-PBA and GA-CDB@Cur were combined through a tumor acidic microenvironment-sensitive borate ester bond to construct AS-PBA/GA-CDB@Cur. *In vitro* studies showed that AS-PBA/GA-CDB@Cur enhanced the cytotoxicity of Cur by reducing the mitochondrial membrane potential and activating apoptotic pathway in AS-PBA/GA-CDB@Cur-treated pancreatic epithelioid carcinoma cells (PANC-1 cells). *In vivo* laboratory studies confirmed that the AS-PBA/GA-CDB@Cur could significantly prolong the retention time of Cur in the tumor tissue and exhibited the strongest anti-tumor effect with no systemic toxicity to PANC-1-bearing nude mice by downregulating Ki 67 and upregulating caspase 3.

**FIGURE 4:**
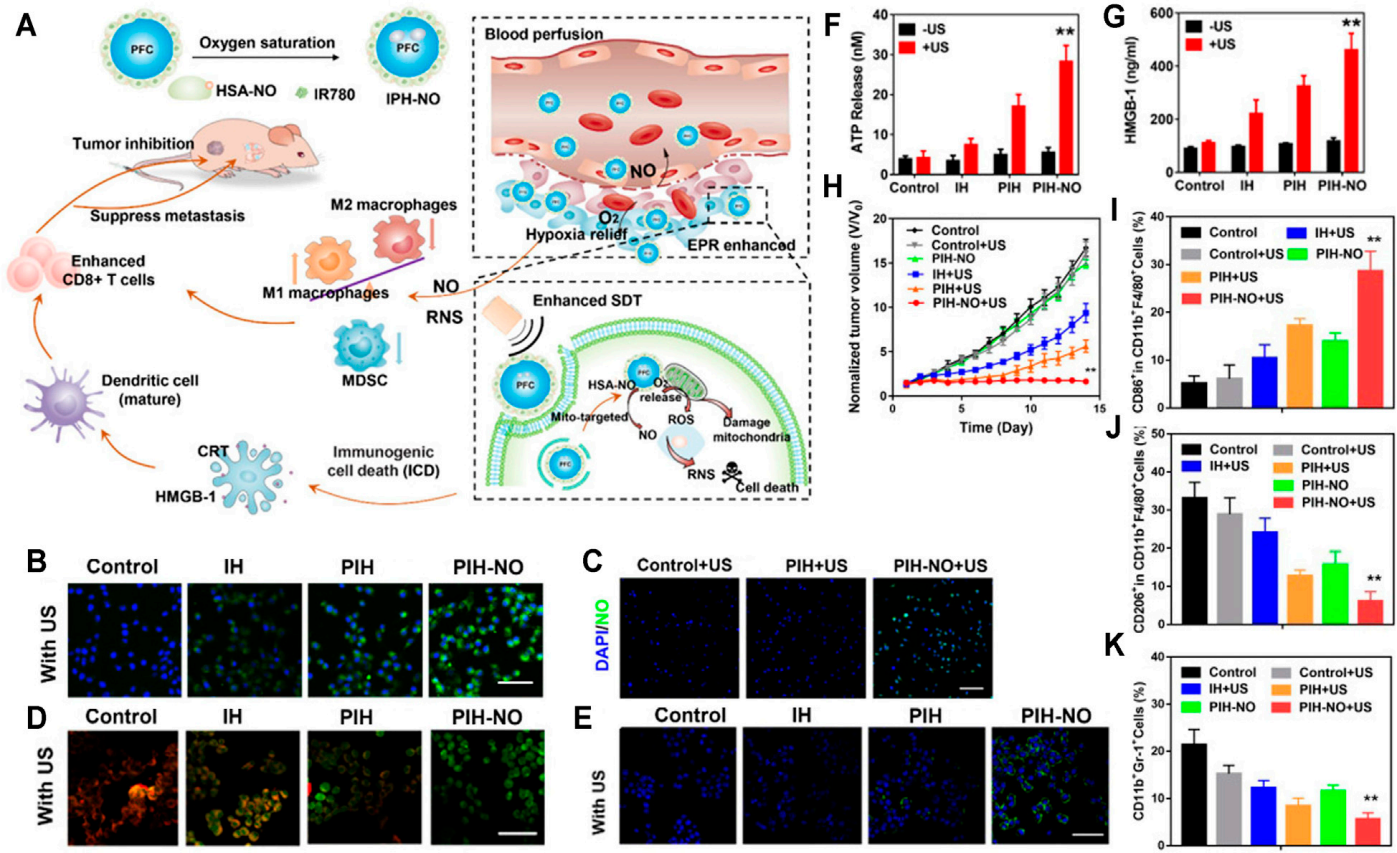
**(A)** PIH-NO for enhanced SDT and immune response. PIH-NO was developed by HSA-NO-loaded PFC and IR780. After injection, it could achieve increased blood perfusion and enhanced EPR effect as well as relieve hypoxia. With ultrasound irradiation treatment, it could quickly release O_2_ and NO to facilitate SDT, destroy mitochondria, and promote DC maturation. The production of NO and RNS could polarize M2 to M1 macrophages and decrease MDSC to reverse immunosuppressive TME. All of the above approaches facilitated CD8^+^ T-cell infiltration to inhibit tumor growth. **(B)** ROS production. **(C)** NO release after different treatments. **(D)** Mitochondrial damage (green fluorescence indicated mitochondrial damage). **(E)** Calreticulin exposure, **(F)** ATP release, and **(G)** HMGB-1 release indicating immunogenic cell death. **(H)** Tumor growth curves after administration. **(I, J)** The proportion of M2 to M1 macrophages. **(K)** Percentages of MDSCs after administration (Ji et al., 2021).

SDT has a great potential to inhibit the growth of tumor cells and trigger antitumor immune responses (Gao G. et, al., 2019; Gong et, al., 2020; Son et al., 2020). However, insufficient blood perfusion, limited oxygen (O_2_) diffusion, and immunosuppressive TME in solid tumors severely limit the anticancer effect of SDT (Jing et al., 2019; Li et al., 2020; Yang et al., 2020). Perfluorocarbon (PFC), a widely used artificial blood substitute with high oxygen-carrying capacity and excellent biocompatibility, has been proven to explosively release O_2_ and significantly enhance PDT under ultrasound (US) (Zhou et al., 2018; Krafft and Riess, 2021). On the other hand, nitric oxide (NO) can effectively inhibit tumor cell respiration and facilitate tumor vessel normalization, which thereby increase the perfusion of blood, alleviate tumor hypoxia, polarize M2 macrophages to the M1 phenotype, and reduce the number of myeloid-derived suppressor cells (MDSCs) (Ramesh et al., 2020). Furthermore, many studies have demonstrated that NO could react with the ROS produced by PDT or SDT, which could generate more powerful oxidant reactive nitrogen species (RNS) to reverse the immunosuppressive TME and achieve more effective antitumor effect (An et al., 2020; Lin et al., 2020; Xiang et al., 2021). Therefore, Ji et al. (2021) constructed a mitochondria-targeting, US-responsive nanoparticle (named PIH-NO), which could deliver O_2_ and NO at the same time to amplify SDT and increase immune response. Briefly, PIH-NO was synthesized with a human serum albumin-based NO donor (HSA-NO) as vehicle to load PFC and IR780 (PFC as the O_2_ donor and IR780 as the sonosensitizer as well as mitochondrial targeting ligand), where HSA-NO is sensitive to US and glutathione for fast NO release. Under US irradiation treatment, PIH-NO displayed a rapid release of O_2_ and NO to alleviate hypoxia and increase blood perfusion, which promoted ROS and peroxynitrite anions (ONOO^−^) production to amplify the anticancer effect of SDT. In addition, the release of O_2_ and NO from PIH-NO at the tumor tissues polarized M2 to M1 phenotype, decreased the quantity of MDSCs, and promoted the maturation of dendritic cells (DCs), which ultimately reversed the immunosuppressive TME and enhanced tumor immunotherapy (Figure 4A). To confirm the ROS production by PIH-NO, 4T1 cells were stained with ROS indicator after being treated with different preparations. As can be seen from Figure 4B, the US plus PIH-NO treatment group showed stronger green fluorescence than the other groups. In a similar way, the release of NO from PIH-NO was also confirmed. As shown in Figure 4C, under US irradiation, more intense green fluorescence was observed in the PIH-NO treatment group. In addition, the damage of mitochondria by PIH-NO was evaluated by using JC-1 (green fluorescence). As shown in Figure 4D, the green fluorescence in the PIH-NO treatment group was more intense than that in other groups. Considering that intracellular ROS can trigger immunogenic cell death, calreticulin exposure (Figure 4E), ATP release (Figure 4F), and HMGB-1 release (Figure 4G), immunogenic cell death has also been tested. The calreticulin exposure, ATP release, and HMGB-1 contents showed trends similar to that of ROS production. Inspired by the *in vitro* therapeutic effect of PIH-NO, Ji et al. evaluated the *in vivo* anticancer effect in 4T1 cell-bearing mice. The tumor growth curve of the US plus PIH-NO treatment group (Figure 4H) proved that PIH-NO could effectively eradicate the solid tumors under US irradiation. Moreover, the proportion of M2 to M1 macrophages (Figures 4I,J) and percentages of MDSCs (Figure 4K) have also been measured *in vivo*. All of the above results indicated that US plus PIH-NO could reverse immunosuppression and enhance tumor immunotherapy.

### DNA Nanostructures

Owing to the unique superiorities of DNA nanostructures including high stability, controllable size and shape, high functional group density, convenient chemical modifications, favorable biodegradability, and biocompatibility, DNA nanostructures are a promising platform for drug delivery and bioimaging. Nowadays, DNA nanostructure-based applications in chemotherapy, PDT, bioimaging, and therapeutic oligonucleotides have been widely reported. Nevertheless, there are still many problems that need to be solved. The pharmacokinetics of DNA nanostructures need to be further studied. More importantly, the biological safety of DNA nanostructures is still unknown. Although DNA nanostructures are biocompatible and biodegradable, whether the foreign DNA sequences in DNA nanostructures can result in harmful gene recombination need to be confirmed (Hu et al., 2019; Duangrat et al., 2020). Compared with mitochondria-targeting nanocarriers, such as liposomes, polymers, and nanoparticles, DNA nanostructures generally have uniform shapes and size, excellent biocompatibility and facile modification, which have been widely used as a novel platform for delivering drugs, genes, and imaging probes (Wang and Ding, 2014; Jiang et al., 2019a; Jiang et al., 2019b). Particularly, tetrahedral DNA nanostructures (TDNs) have shown excellent controlled drug release and drug-loading abilities. Moreover, functional moieties (including peptides, antibodies, and targeting aptamers) can be precisely decorated on TDNs to obtain targeting property (Liang et al., 2014; Li W.-Q. et al., 2017; Zhang et al., 2020). Therefore, these superiorities make them more attractive to create various nanosystems for targeted drug delivery. Yan et al. (2020) constructed a novel nanoplatform based on TDNs, which enabled DOX to specifically reach the mitochondria. For mitochondria-targeted delivery, various numbers of KLA were connected to DNA and then self-assembled to form KLA-modified TDNs. Finally, DOX was loaded into KLA-modified TDNs. Among them, the 3KLA-modified TDNs (3KLA-TDNs/DOX) exhibited the strongest mitochondria-targeting capacity, programmed apoptosis pathway activation, and tumor cytotoxicity by facilitating abundant release of cytochrome c; upregulating expression levels of caspase-3, caspase-9, p21, and p53; and downregulating the anti-apoptotic Bcl-2 protein expression and upregulating pro-apoptotic Bax.

### Polymeric Nanomicelles

Polymeric nanomicelles are well-organized supramolecular structures formed by self-assembly of amphiphilic polymers in aqueous media, whose hydrophobic “inner cores” are responsible for loading lipophilic drugs and controlling the drug release behavior, and whose hydrophilic “outer shells” are in charge of improving the pharmacokinetic properties in blood circulation. Polymeric nanomicelles have been proven to be a promising nanodrug platform owing to their advantages including good biocompatibility, economy, and ease of further modification to facilitate targeted delivery. In addition, nanosystems-based biological imaging, especially fluorescent nanomicelles, emerges as an ideal cancer screening tool. However, the clinical application of fluorescent nanomicelles is strictly limited, because of drug leakage in blood, nonspecific toxicity, and rapid clearance by plasma (Li et al., 2019; Tawfik et al., 2020). Although TPP-conjugated derivatives possess both mitochondrial targeting and mitochondrial damaging functions just like TPP, those lipophilic cation derivatives could be eliminated in blood quickly (Khatun et al., 2017; Liu et al., 2018; Song et al., 2018). Chondroitin sulfate (CS), as an anionic and a CD44 acceptor, can be employed to modify nanoparticles, and this can prolong blood circulation as well as endow an active targeting ability to the cell membrane (Liu M. et al., 2018; Lee et al., 2020; Li et al., 2021). Herein, Zhang et al. (2020) developed pH/redox dual-sensitive, mitochondria/cell membrane synergic targeting nanomicelles for mitochondrial targeted therapy. Briefly, the nanomicelles loaded with DOX were based on TPP-decorated poly(ethylene glycol) (PEG)-poly (D, L-lactide) (PLA) copolymers (TPP-PEG-ss-PLA). Among them, TPP-PEG-ss-PLA copolymers were constructed by using disulfide bonds, which could self-assemble into nanomicelles and promote quick release of DOX when the nanomicelles disassembled in GSH-triggered redox responsiveness. To prolong the half-life of nanomicelles and facilitate the endocytosis of cancer cells, positively charged TPP-PEG-ss-PLA were transformed into negatively charged *via* modifying the nanomicelles with CS. As long as the vehicles reached lysosomes/endosomes, the CS layer (negatively charged) could fall off when the pH changed from 7.4 to 5.5, facilitating the expose of the TPP and charge reversal on the surface of nanomicelles. In their study, DOX-loaded CS/TPP-PEG-ss-PLA nanomicelles exhibited significant synergistic antitumor effect owing to overproduction of ROS in the mitochondria induced by TPP and mitochondrial DNA and nuclear DNA damage induced by DOX.

Pluronic P85 (P85), a widely used surfactant, has been proven to reverse drug resistance in previous studies (Hong et al., 2013; Alakhova and Kabanov, 2014; Fang et al., 2016). Wang et al. (2021) synthesized a TPP derivate (P85-SS-TPP, P-SS-T) with positive charge, where P85 was combined with TPP *via* a disulfide bond to promote quick release of the drugs when the nanomicelles disassembled in GSH-triggered redox responsiveness. Then, a weak acid-sensitive dimethylmaleic anhydride (DA) protection group with negative charge was applied to decorate P-SS-T to get DA-P-SS-T (negatively charged), where DA could extend the blood circulation and enhance the cellular uptake of P-SS-T. In their study, PTX-loaded DA-P-SS-T nanomicelles (DA-P-SS-T/PTX), which were pH-responsive, charge-reversible, and redox-responsive, showed great potential to reverse drug resistance and exhibited enhanced antitumor effect in lung cancer, owing to the decreased expression level of P-gp based on the reduction of mitochondrial ATP as well as PTX-loaded mitochondrial DNA damage and microtubule damage.

D-α-tocopheryl polyethylene glycol succinate (TPGS) has been widely applied as a carrier material owing to its capability of downregulating the expression of P-gp for overcoming multidrug resistance (MDR) and good amphiphilicity in favor of improving the penetration and circulation time of drug delivery systems (DDS) (Dintaman and Silverman., 1999; Shafiei-Irannejad et al., 2018; Yang et al., 2018; Yan et al., 2021). On the other hand, positively charged TPP can penetrate the mitochondrial membranes and thereby specifically reach the highly negatively charged mitochondria to facilitate mitochondria targeting. Zhang et al. (2017) designed a new, trackable, mitochondria-targeting drug delivery platform (CQDs-TPGS-TPP) on the basis of the self-assembly, TPP-decorated TPGS nanomicelles (TPGS-TPP), and fluorescent carbon quantum dots (CQDs), where CQDs were used as fluorescent indicators to realize real-time monitoring of nanomicelle penetration and localization inside tumor cells. Then, DOX was loaded into CQDs-TPGS-TPP nanomicelles to obtain CQDs-TPGS-TPP/DOX. In their study, the enhanced penetration efficiency of CQDs-TPGS-TPP/DOX was clearly observed in MCF-7/ADR three-dimensional multicellular spheroids (MCs). The calculated resistant index (RI) of CQDs-TPGS-TPP/DOX (7.16) was obviously decreased compared with the free DOX (66.23), while treating drug-resistant MCF-7. CQDs-TPGS-TPP/DOX induced more apoptosis of MCF-7/ADR and showed an excellent MDR reversal capacity in MCF-7/ADR MCs, attributed to the additive effects of P-gp downregulation from TPGS, mtDNA damage, and nucleus damage from DOX.

Although many mitochondria-targeting nanoparticles have been used to enhance cancer treatment, the leakage of drugs in cytoplasm and lysosome is challenging for all researchers when the therapeutic nanoparticles get inside the cancer cells (Wong and Choi, 2015; Xiong et al., 2016; Huang et al., 2021). As we know, the pH value of tumor cell mitochondria is approximately 8.0 (Matsuyama et al., 2000; Zhao et al., 2020). Thus, Tan et al. (2018) constructed a mitochondrial alkaline-responsive drug delivery system loading an acidic drug, which could not only facilitate fast drug release in mitochondria to improve the anticancer efficiency, but also decrease the nonspecific toxicity observably by means of reducing the drug leakage in the neutral cytoplasm and acidic lysosome. In their study, they used lipophilic cation CTPP (a kind of TPP derivative) to decorate glucolipid-like conjugates (chitosan-stearic acid copolymer, CSOSA) as a mitochondria-targeting platform, which could self-assemble into nanomicelles (CTPP-CSOSA). Then, celastrol (Cela), a kind of weakly acidic drug, which could react on mitochondrial respiratory chain (MRC) complex I and trigger ROS accumulation, was loaded into CTPP-CSOSA to obtain CTPP-CSOSA/Cela. It confirmed that CTPP-CSOSA/Cela showed fast drug release in mitochondria with decreased drug leakage in the lysosome and cytoplasm, induced remarkably intensive ROS levels, had an enhanced accumulation in tumor tissue, and showed significant antitumor effect by decreasing mitochondrial membrane potential, promoting cytochrome c release, and upregulating caspase 3 and caspase 9.

Hsp90 can quickly fix thermal damage to proteins thus causing heat resistance of tumor cells (Schopf et al., 2017; MoránLuengo et al., 2019). Therefore, synergistic application of Hsp90 inhibitor and mild-temperature photothermal therapy (MT-PTT) can effectively inhibit Hsp90 expression in tumors, overcome tumor thermal resistance, and realize efficient mild-temperature heating effects without thermal damage to surrounding tissues (Wang et al., 2016; Yang et al., 2019a; Gao et al., 2019; Shao et al., 2020). Specifically, BIIB021, an excellent candidate of Hsp90 inhibitor, has demonstrated promising efficacy in laboratory studies and is now included in clinical research in gastrointestinal stromal tumors and Kaposi sarcoma (Chen et al., 2012; Dickson et al., 2013). Zhang et al. (2021) developed mitochondria-targeting nanomicelles containing BIIB021 (named PEG-IR780-BIIB021), which could not only selectively accumulate in tumors without damaging the surrounding normal tissues but also exhibit strong mild-temperature photothermal therapy without tumor thermal resistance under laser irradiation in MCF-7 cell-bearing mice by regulating Cyt-C, Bcl-2, caspase-9, and Bax.

Although nanoparticles provide a platform for the *in vivo* applications of mitochondria-targeting PDT, their poor cancer cell membrane-targeting ability is not good for further mitochondrial targeting only (subcellular targeting) by tumor cells. Peng et al. (2020) developed a sequential-targeting (tumor cell membrane and mitochondrial targeting) delivery system (named C6-loaded BioPEGDMA@TPPM) on the basis of the core-shell structure. Among them, mitochondria-targeting, chlorin e6-loaded cationic amphiphilic copolymer (C6-loaded TPPM) served as the core, and tumor cell membrane-targeting, pH-sensitive charge reversal layer based on the 2,3-dimethylmaleic anhydride (DMA)-decorated Biotin-PEG_4000_-NH_2_ (BioPEGDMA) served as the shell. In their study, Ce6-loaded BioPEGDMA@TPPM was firstly cell membrane-targeted and effectively accumulated in the tumor tissue with the aid of Biotin moiety. Then, Ce6-loaded BioPEGDMA@TPPM was mitochondria-targeted facilitated by TPP. Among them, TPP exposed through acid-triggered charge reversal from negative to positive, which ultimately enhanced the PDT efficacy of Ce6 and simultaneously stimulated immune responses by upregulating expression of TNF-α, IFN-γ, and CD3^+^ in tumor tissues, facilitating activation of DCs, CD3^+^/CD4^+^, and CD3^+^/CD8^+^ T lymphocytes in lymph glands as well as tumor tissues.

**FIGURE 5:**
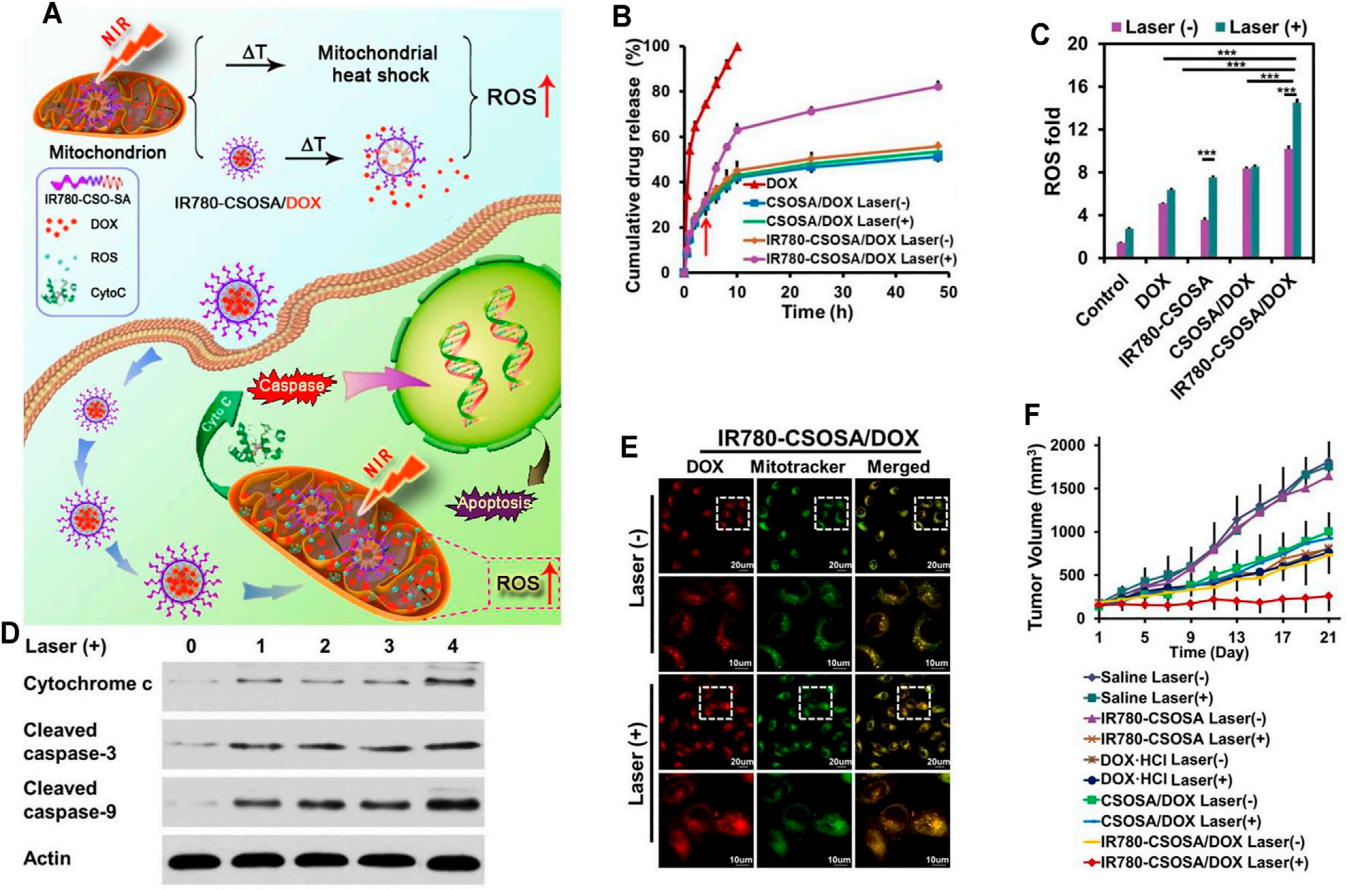
**(A)** The NIR-triggered DDS with mitochondria-sensitive drug release and heat shock capabilities. Under NIR, IR780-CSOSA/DOX nanomicelles selectively targeted mitochondria and achieved photothermal conversion, which photothermally triggered DOX release and heat shock in mitochondria, leading to a cascading effect on ROS burst to achieve amplified therapeutic efficacy. **(B)** NIR-triggered DOX release in PBS (pH 6.8). **(C)** ROS levels in MCF-7. **(D)** Levels of apoptosis proteins in MCF-7. 0: control, 1: DOX, 2: CSOSA/DOX, 3: IR780-CSOSA, 4: IR780-CSOSA/DOX. **(E)** DOX release into the mitochondria in MCF-7. **(F)** Tumor growth curves after administration (Tan et al., 2019).

The sufficient drug concentration in mitochondria is an indispensable premise for nanoparticles contributing to better anticancer effects (Wen et al., 2016; Zielonka et al., 2017). Therefore, mitochondrial-triggered drug release in mitochondria is urgently essential (Biswas and Torchilin, 2014; Joo, 2021). IR780 (IR-780 iodide) with lipophilic cation characters is a NIR-sensitive lipophilic photothermal agent with mitochondria-targeting feature as well as real-time diagnosis and imaging functions (Peng et al., 2011; Shih et al., 2017; Tan et al., 2017; Xia et al., 2019). Tan et al. (2019) chose IR780 to modify CSOSA, named IR780-CSOSA, which could self-assemble into nanomicelles. Then, DOX was loaded into IR780-CSOSA to obtain IR780-CSOSA/DOX. Specifically, with laser irradiation treatment, the photothermal conversion and resulting hyperthermia of IR780-CSOSA not only could damage the hydrophobic interaction between DOX and the core of nanomicelles, triggering amazing nanomicelles’ swelling to facilitate the fast liberation of DOX in mitochondria so as to elevate the level of ROS, but also could trigger mitochondria-specific heat shock to facilitate the rapid evolution of ROS at the same locus to eliminate tumor cells in a superefficient way, which consequently led to precise and superadditive cancer chemo-phototherapy (Figure 5A). As mentioned above, IR780-CSOSA/DOX nanomicelles showed an obvious photothermal feature under NIR. In the first 4 h, IR780-CSOSA/DOX showed a slow-release behavior. Nevertheless, IR780-CSOSA/DOX showed rapid DOX release triggered by NIR after 4 h of incubation (Figure 5B). Figure 5E shows that there was a good overlap between the green fluorescence and red fluorescence in the IR780-CSOSA/DOX plus NIR treatment group, indicating that NIR could specifically and effectively induce drug release from IR780-CSOSA into the mitochondria. To elucidate the underlying mechanism of *in vitro* antitumor activity of IR780-CSOSA/DOX, Tan et al. measured the expression levels of related proteins. As shown in Figure 5C, ROS levels were highest in the IR780-CSOSA/DOX plus NIR treatment group, which indicated that NIR could significantly strengthen the ROS production of IR780-CSOSA/DOX. On the other hand, the levels of cytochrome c and cleaved caspase-3 and -9 were tested. As can be seen from Figure 5D, cytochrome c and cleaved caspase-3 and -9 were significantly increased in the IR780-CSOSA/DOX plus NIR treatment group. Inspired by the *in vitro* therapeutic effect of IR780-CSOSA/DOX, Tan et al. evaluated the *in vivo* anticancer effect in MCF-7 cell-bearing mice. The tumor growth curve demonstrated that the IR780-CSOSA/DOX plus NIR treatment group had the best inhibitory effect on tumor growth (Figure 5F).

CDT is a novel therapeutic strategy based on the weakly acidic microenvironment of tumor as reaction conditions, overexpressed H_2_O_2_ in tumor sites as reaction raw materials, and ferrous ion (Fe^2+^) as catalyst, which can trigger Fenton or Fenton-like reaction in tumor cells and catalyze H_2_O_2_ to produce hydroxyl radical (·OH) and ROS so as to induce tumor cell apoptosis (Nie et al., 2019; Shen et al., 2020; Tian et al., 2020). On the other hand, lonidamine, an antiglycolytic drug, has been shown to interfere tumor energy metabolism by inhibiting hexokinase (Nath et al., 2016; Cheng W. et al., 2019; Cohen-Erez et al., 2019). However, poor solubility in water and nonspecific toxicity limited its clinical application. Therefore, Sun M. et al. (2021) constructed a multifunctional mitochondria-targeting prodrug nanosystem based on polylysine, which synergistically utilized the metabolic inhibition achieved by lonidamine and CDT realized by Fenton reaction to amplify antitumor treatment and overcome drug resistance. First of all, TPP and lonidamine were grafted onto polylysine by ester bonds to form mitochondria-targeting prodrug (T-Prodrug), which could self-assemble into nanomicelles. Then, ferrocene and glucose oxidase (GOx) were crosslinked onto the surfaces of T-Prodrug nanomicelles to obtain ferrocene and Gox-loaded T-Prodrug nanomicelles (FG/T-Nanoprodrug) for synergistic CDT. Among them, Gox could provide cancer cells with high concentration of H_2_O_2_ to enhance CDT. In an *in vitro* study, FG/T-Nanoprodrug showed efficient mitochondria-targeting feature and energy metabolism inhibition in A549/DDP. Moreover, due to the reduced glucose consumption induced by suppressed energy metabolism, Gox and ferrocene could generate excess ROS to amplify CDT, which consequently enhanced the permeability of mitochondria, facilitated the release of cytochrome c, and finally triggered the autophagy of mitochondria. In an *in vivo* study, FG/T-Nanoprodrug also achieved significantly enhanced tumor growth inhibition owing to the superadditive effects of autophagy and energy metabolism inhibition.

## Conclusion and Future Prospect

Mitochondria, a kind of subcellular organelle, play crucial roles in cancer cells as an energy source and as a generator of reactive substrates, which concern the survival, invasion, proliferation, and drug resistance of cancer. Thus, mitochondrial-targeting DDS-based nanosystems could be novel strategies for enhanced cancer treatment. In this review, we summarize the very latest developments of mitochondria-targeting nanomedicines in cancer treatment as well as focus on designing multifunctional mitochondria-targeting nanosystems based on the latest nanotechnology. As we discussed above, numerous multifunctional mitochondrial-targeting DDS, such as nanoparticles, liposomes, and nanomicelles, have been constructed for chemotherapy, PDT, PTT, immunotherapy, combined therapy, etc., to achieve better therapy efficacy. The mechanism of those multifunctional mitochondrial-targeting nanomedicines is to induce intrinsic cell apoptosis and/or cell necrosis mediated by interfering with energy metabolism, increasing exogenous ROS, damaging mtDNA, destroying redox homeostasis, regulating mitochondrial proteins, inducing mitophagy, or triggering the immune response. There is no doubt that multifunctional mitochondrial-targeting-based nanoplatforms provide a new field in nanotechnology for precise subcellular targeting.

Nevertheless, we must recognize that there is still a long way to go when multifunctional mitochondrial-targeting nanomedicines can be used in clinic. First of all, the most important thing is biosafety, as is known to all that both biosafety and effectiveness are the essential safeguards that nanomedicines can be included in clinical application. Therefore, it is urgently needed to construct more biocompatible nano-vehicles through practical design. For instance, polydopamine (PDA)- or tetrahedral DNA (TDNs)-based nanomedicines have been used for mitochondrial targeting recently, which showed excellent biocompatibility. In addition to promoting biosafety, the amount of nanomedicines that can reach tumor cells and eventually accumulate in mitochondria is still a hair off a bull’s back. Therefore, a multistage targeted DDS capable of realizing both tumor cell membrane and mitochondria targeting is highly desirable. For instance, tumor cell-specific ligands such as Biotin, cRGD, H peptide, and modified mitochondrial-targeting nanomedicines have shown enhanced cellular uptake and antitumor effects. Last, the insufficient drug concentration in mitochondria is another obstacle that hinders mitochondrial-targeting nanomedicines from contributing better anticancer effects. To overcome this challenge, utilizing the unique alkaline microenvironment (pH 8.0) of mitochondria can realize fast drug release in mitochondria; in addition, with the aid of external stimulation, such as NIR, laser irradiation, and US, the drug release of nanomedicines in mitochondria can also be precisely controlled. All in all, it would be highly desirable to design “smart, multifunctional, mitochondria targeted-delivery” nanomedicines for precise cancer treatment, which need more in-depth study and exploration by researchers from different fields.

In addition to cancer therapy, mitochondria-targeted nanomedicines also offer a promising hope for the treatment of other mitochondria-related diseases, such as Parkinson’s and Alzheimer’s disease. It should be pointed out that we may face bigger challenges in breaking through the blood–brain barrier and delivering drugs to targeting sites effectively for these diseases. Altogether, although there are many challenges and problems that need to be solved in designing mitochondria-targeting-based nanomedicines, we firmly believe that these difficulties will ultimately be overcome and more stirring breakthroughs will be achieved in the near future.
